# Gothenburg Breast reconstruction (GoBreast) II protocol: a Swedish partially randomised patient preference, superiority trial comparing autologous and implant-based breast reconstruction

**DOI:** 10.1136/bmjopen-2024-084025

**Published:** 2024-07-17

**Authors:** Emma Hansson, Jonas Löfstrand, Camilla Larsson, Alexandra Uusimaki, Karolina Svensson, Anna Ekman, Mikael Svensson, Anna Paganini

**Affiliations:** 1 Department of Plastic Surgery, Institute of Clinical Sciences, Sahlgrenska Academy, University of Gothenburg, Gothenburg, Sweden; 2 Region Västra Götaland, Sahlgrenska University Hospital, Department of Plastic and Reconstructive Surgery, Gothenburg, Sweden; 3 Johanna, Regional branch of the Swedish Breast Cancer Association, Gothenburg, Sweden; 4 Department of Public Health and Community Medicine, Institute of Medicine, Sahlgrenska Academy, Gothenburg University, Gothenburg, Sweden; 5 Department of Diagnostics, Acute and Critical Care, Institute of Health and Care Sciences, Sahlgrenska Academy, Gothenburg University, Gothenburg, Sweden

**Keywords:** PLASTIC & RECONSTRUCTIVE SURGERY, Breast surgery, Randomized Controlled Trial

## Abstract

**Introduction:**

Although breast reconstruction is an integral part of breast cancer treatment, there is little high-quality evidence to indicate which method is the most effective. Randomised controlled trials (RCTs) are generally thought to provide the most solid scientific evidence, but there are significant barriers to conducting RCTs in breast reconstruction, making both recruitment and achieving unbiased and generalisable results a challenge. The objective of this study is to compare implant-based and autologous breast reconstruction in non-irradiated patients. Moreover, the study aims to improve the evidence for trial decision-making in breast reconstruction.

**Methods and analysis:**

The study design partially randomised patient preference trial might be a way to overcome the aforementioned challenges. In the present study, patients who consent to randomisation will be randomised to implant-based and autologous breast reconstruction, whereas patients with strong preferences will be able to choose the method. The study is designed as a superiority trial based on the patient-reported questionnaire BREAST-Q and 124 participants will be randomised. In the preference cohort, patients will be included until 62 participants have selected the least popular alternative. Follow-up will be 60 months. Embedded qualitative studies and within-trial economic evaluation will be performed. The primary outcome is patient-reported breast-specific quality of life/satisfaction, and the secondary outcomes are complications, factors affecting satisfaction and cost-effectiveness.

**Ethics and dissemination:**

The study has been approved by the Swedish Ethical Review Authority (2023-04754-01). Results will be published in peer-reviewed scientific journals and presented at peer-reviewed scientific meetings.

**Trial registration number:**

NCT06195865.

**STRENGTHS AND LIMITATIONS OF THIS STUDY**

- This protocol uses a partially randomised patient preference trial design to compare different techniques in breast reconstruction.
- The protocol includes studies within a trial to explore the research methodology further.
- The protocol’s outcomes measures include outcomes important to patients, professionals and society, such as patient-reported outcomes, complications and cost-effectiveness.
- The protocol’s conceptual risks include difficulty recruiting participants, especially to the randomised arm, and a low adherence and retention.
- The protocol includes patients from a single country, which might limit the generalisation to different healthcare systems.

## Introduction

### Background and rationale

 Breast reconstruction is an integral part of modern breast cancer treatment.^1 2^ Nevertheless, evidence for the effectiveness of breast reconstruction methods is lacking with respect to increasing quality of life and achieving high patient satisfaction, with a low complication rate and societal economic costs. The low evidence is reflected in the varying guidelines for breast reconstruction and unequal access to different methods that have been seen in a European study,^3^ as well as in a report published by the Swedish Breast Cancer Association.^4^ Techniques for breast reconstruction can roughly be divided into two categories: *autologous* and *implant-based* techniques. Three systematic reviews^5^,^7^ have concluded that patients seem to have a higher breast-related quality of life when reconstructed with autologous techniques compared with implant-based techniques. However, most of the included studies were retrospective, non-randomised, did not correct for other factors that might affect satisfaction, and had a short follow-up. Moreover, few high-quality studies compare long-term cost-effectiveness^8^ and the long-term need for revisions, corrections, and donor-site consequences. All of these factors are essential to create evidence-based guidelines, prioritise the usage of healthcare resources and to give the patients information on which they can base decisions of breast reconstruction. There are no ongoing trials registered in ClinicalTrials.gov comparing different categories of breast reconstruction technique head to head (https://clinicaltrials.gov/search?cond=breastreconstruction (search performed 22 July 2023)).

Randomised controlled trials (RCT) are generally thought to provide the most solid scientific evidence for treatment effects. However, there are barriers to conducting RCTs in breast reconstruction, making both recruitment and achieving unbiased and generalisable results a challenge.^9 10^ First, an RCT requires that there is solid uncertainty about which method achieves the best results. In the case of breast reconstruction, the operating surgeon must not prefer one method to another (*theoretical equipoise*)^11^ as this could result in both biased recruitment as well as biased outcomes if the included patients receive biased preoperative and postoperative information.^9^ The patient must also not have preformed ideas and clear preferences regarding the different methods (*principle of indifference*)^11^ based on, for example, other patients, patient organisations, and the media, as this also affects the recruitment and the results. Patients’ preferences can affect the external validity if a standard treatment, for example, implant-based breast reconstruction in non-irradiated patients,^12^ is compared with an alternative treatment, for example, autologous reconstruction, as only patients who prefer autologous reconstruction are likely to accept randomisation. Patients’ preferences could also reduce internal validity as randomisation to the (non-) preferred strategy could affect both adherence to the protocol (*reluctant acquiescence phenomenon*)^13^ and outcome. All these factors would lead to results that are not generalisable to the clinical population. This risk of bias and low internal and external validity illustrates why an RCT can be an inappropriate study design when comparing different categories of breast reconstruction.

A PubMed search on breast AND reconstruction, limited to RCTs, yields 419 results (10.07.2023).^14^ One-hundred and nine of them are RCTs concerning some aspect of breast reconstruction. The majority compared surgical variation within the same category of breast reconstruction technique, for example, one versus two stages or mesh versus no mesh in implant-based breast reconstruction and preoperative imaging versus no preoperative imaging in autologous breast reconstruction. Only four studies compare different categories of breast reconstruction techniques head to head^15^,^18^ and they all illustrate the aforementioned challenges with RCTs in breast reconstruction. For example, in the RCT performed in our department, the Gothenburg Breast Reconstitution trial (GoBreast),^18 19^ preintervention dropouts rates after randomisation varied between 12.5% and 23% in different groups, due to either the patient’s or surgeon’s preferences.

The study design partially randomised patient preference trial (RPPT) is an approach to diminish the impact of patients’ preferences, facilitate recruitment, increase patient centricity, decrease the risk of excluding large patient groups and make the results more generalisable to the clinical population, when preference-sensitive interventions are compared.^20^ In an RPPT, patients with a clear preference are treated accordingly and patients without a distinct preference are randomised in the traditional way. The RPPT design enables a more efficient inclusion of participants, and a clinically more representative study population, while maintaining a high external and internal validity.^20^ GoBreast II will mark the first use of an RPPT design to evaluate breast reconstruction methods.

### Choice of comparators

There is a myriad of different surgical options in breast reconstruction, such as different meshes and implants, as well as different pedicled or free flaps, but there are two main categories: implant based or autologous breast reconstruction. The two main categories are compared in this study.

### Research hypotheses

It is hypothesised that

- patients are more satisfied with the reconstructed breast/s when an autologous deep inferior epigastric perforator (DIEP) flap is performed.
- although a ‘simpler procedure’, implant-based reconstruction entails a higher total number of operations and revisions long-term, compared with autologous DIEP-flap.
- although a procedure that is more costly for the healthcare system when it is performed, an autologous DIEP-flap is more cost-effective for society in the long-term perspective, due to the long-term effects and consequences of implants.

### Study objectives

The main overall purpose of breast reconstruction is to increase the woman’s quality of life, both physically and psychosocially. Therefore, the primary objective/outcome is to compare the two methods regarding patient-reported breast-specific quality of life and satisfaction. These measures are also part of the core outcome set for breast reconstruction developed by patients and professionals.^21^

The secondary objectives are to compare the two methods regarding complications, unplanned operations, corrections, cost-effectiveness and factors that might affect the primary outcome. Other secondary objectives are to improve the evidence for trial decision-making in breast reconstruction to improve the methodological design and process of future studies by means of a study within a trial (SWAT).^22^

### Trial design

**Figure 1:**
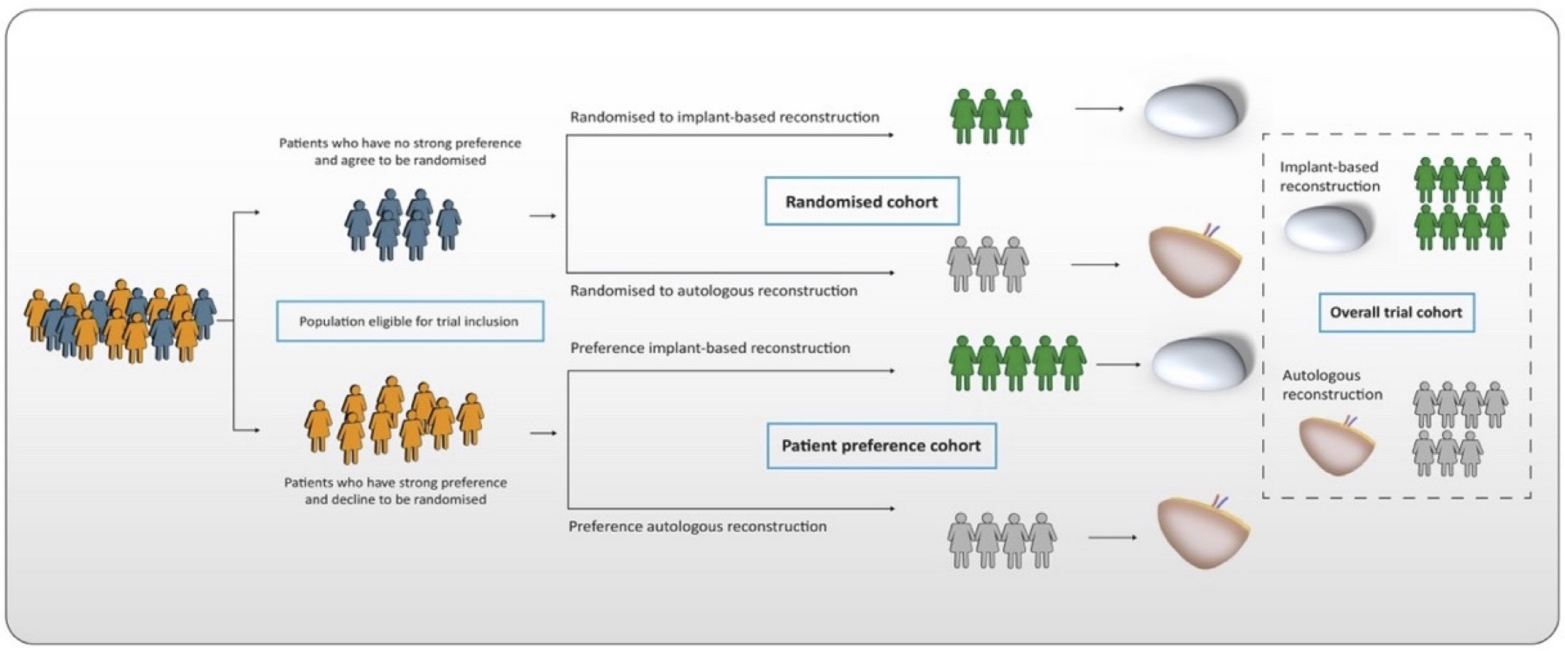
The partially randomised patient preference trial design (RPPT) and allocation of the patients. Figure by Niclas Löfgren, Department of Plastic and Reconstructive Surgery, Sahlgrenska University Hospital.

GoBreast II is a partially RPPT with a superiority framework. Participants who accept randomisation will be allocated to one of the two methods. Participants who do not accept randomisation will be operated with their preferred method. Thus, the study has two cohorts: one randomised and one patient preference (figure 1). The trial is a single-centre study conducted at a university hospital in Sweden. It has embedded qualitative and health economic research questions.

## Methods and analysis

### Reporting and preregistration

This protocol is reported in accordance with the Standard Protocol Items: Recommendations for Interventional Trials (SPIRIT) statement 2013^23^ (online supplemental appendix 1), including the SPIRIT-PRO extension^24^ (online supplemental appendix 2). The trial was registered at (ClinicalTrials.Gov identifier NCT06195865).

### Study setting

The study will be performed at Sahlgrenska University Hospital in Gothenburg, Sweden, where the Department of Plastic Surgery currently performs about 350 breast reconstructions yearly, both in the immediate and the delayed setting. In the catchment area of Sahlgrenska University Hospital, all patients diagnosed with breast cancer who have had or will receive a mastectomy and are considering a breast reconstruction are referred to this department. Among the referrals, potentially eligible participants will be invited to consider participation in the trial. According to the current Swedish guidelines,^12^ non-irradiated patients are offered mainly implant-based breast reconstruction and irradiated patients autologous techniques. The Swedish healthcare system is a publicly funded welfare-type healthcare system with a strong emphasis on equal access.

### Population

#### Recruitment and inclusion and exclusion criteria

**Figure 2:**
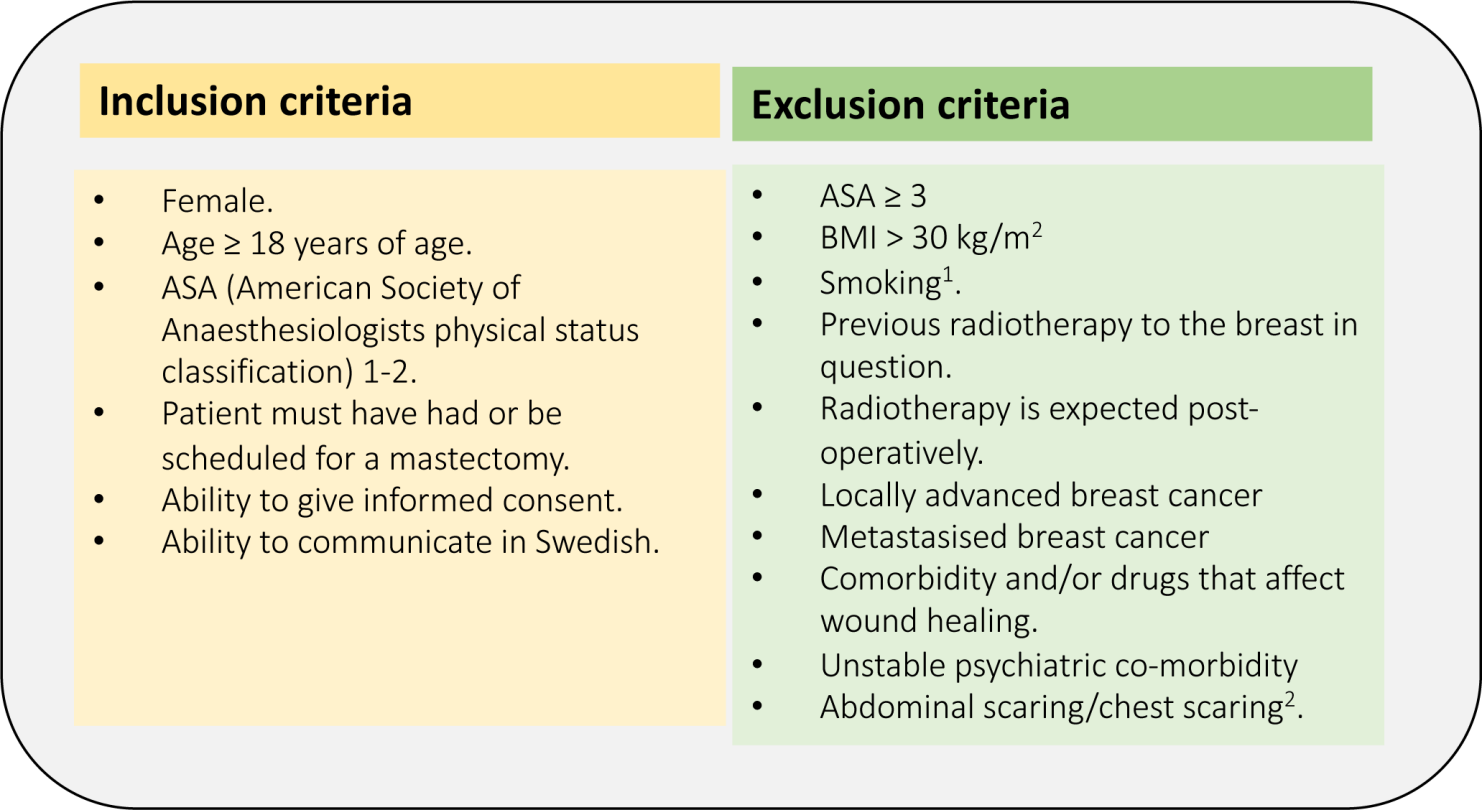
Inclusion and exclusion criteria. ^1^Immediate breast reconstruction: to stop smoking when they are informed about the diagnosis and abstain from smoking at least 6 weeks postop. Delayed breast reconstruction: abstain from smoking 6 weeks preoperatively and 6 weeks postoperatively. ^2^Making a DIEP-flap impossible/an implant-based reconstruction unsuitable. ASA, American Society of Anesthesiologists classification; BMI, body mass index; DIEP, deep inferior epigastric perforator flap.

Among the referrals to the department, potentially eligible participants will be invited to consider participation in the trial. Inclusion and exclusion criteria are given in figure 2.

#### Sample size

**Figure 3:**
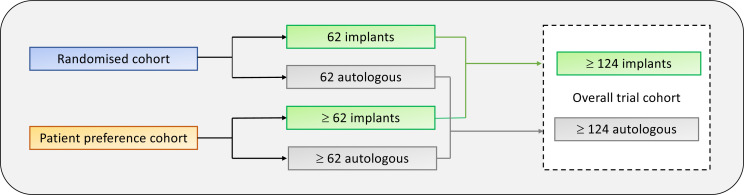
Sample sizes in the randomised and patient preference cohort.

The study is s a superiority trial based on the BREAST-Q domain *Satisfaction with the breast/s*.^25^,^27^ The clinically meaningful difference in BREAST-Q was set to 10 points. There are no anchor-based minimal important differences (MIDs) published for BREAST-Q, but the distribution-based MID is 4 for satisfaction with the breast/s.^28^ The SD was set to 18, as calculated according to US norms.^29^ If power is set to 0.80 and alpha to 0.05, the case-to-control ratio is 1, and a 20% dropout rate is expected, 62 patients are needed in each group (https://riskcalc.org/samplesize/). In the randomised cohort, 124 participants will be randomised 1:1. In the preference cohort, patients will be included until 62 participants have selected the least popular alternative (figure 3). This will result in an overall trial cohort with a minimum of 124 participants in each arm and enough participants in the subgroups *randomised* and *patient preference* to allow for analyses of differences.

#### Uniform preoperative counselling

Uniform counselling is crucial in the RPPT design.^20^ All patients eligible for inclusion in the study will be counselled using the *Patients’ Expectations and Goals of reconstruction. Assisting Shared Understanding of Surgery (PEGASUS*) tool^30 31^ to decide whether they want a breast reconstruction. The tool forms a basis for a patient-centred dialogue around breast reconstruction. An implementation study using PEGASUS in a Swedish context is currently being performed in our department. Following the PEGASUS session, an appointment with a plastic surgeon skilled in both implant-based and autologous breast reconstruction will be scheduled for more technical counselling regarding the two reconstructive options. The information will be standardised for the study.

### Interventions

#### Mastectomy

The mastectomies will be performed by general surgeons (breast surgeons). In case of an immediate breast reconstruction, a skin-sparing mastectomy will be performed. The nipple-areolar complex (NAC) will be preserved if it is oncologically safe. In case of an immediate breast reconstruction, a Wise pattern skin resection will be made in ptotic; otherwise, a submammary incision, or vertical incision if the NAC has to be removed, will be performed. Delayed breast reconstruction will be performed following a simple mastectomy. All reconstructions will be performed by consultant plastic surgeons with a subspecialty in breast reconstruction and a minimum of 5 years of independent experience with the used techniques.

#### Autologous breast reconstruction: DIEP-flap

All DIEP-flaps are performed by plastic surgeons according to the standard of care of the department. In summary, it is performed as a cutaneous-adipose flap, without muscle, and if possible anastomosed to the internal thoracic artery and vein. If needed, the superficial epigastric vein is anastomosed to the cephalic vein through a small incision in the axillary fold. If possible, the flap is buried, when an immediate breast reconstruction is performed. In delayed breast reconstruction, a skin island is inserted between the submammary fold and the old mastectomy scar.

#### Implant-based breast reconstruction

All implant-based breast reconstructions are performed by plastic surgeons according to the standard of care of the department. In immediate breast reconstruction, a subpectoral pocket is created, and the inferior-medial and inferior attachments of the major pectoral muscle are released. If a permanent implant (CPG, Mentor Worldwide LLC, California, USA) is used, a synthetic TIGR Matrix Surgical Mesh (Novus Scientific, Uppsala, Sweden)^32 33^ is sutured to the inferior border of the pectoral muscle and to the chest wall corresponding to the inframammary fold and lateral border of the implant pocket; hence, a dual plane approach is applied. *The Gothenburg TIGR/Veritas Study*^33^,^35^ comparing a biological and a synthetic mesh in immediate breast reconstruction demonstrated that the synthetic mesh is superior to the biological regarding complications and is equivalent regarding patient satisfaction. Therefore, the synthetic mesh has become a standard of care and will be used in the present study. If a tissue expander (CPX4 or Siltex Becker, Mentor Worldwide LLC) is used, a m. serratus pocket is created to block the expander laterally to achieve a muscle-covered device. A temporary expander is exchanged for a permanent implant about 3 months after the initial operation.

### Criteria for discontinuing or modifying allocated interventions for a given trial participant

Trial participants have the right to withdraw from the study at any time without any consequence. Before the reconstruction, patients can change from the randomised cohort to the preference cohort should they change their wishes. Their data will then be analysed according to their change. If the patient regrets her choice of cohort after her reconstruction, she will be included in the analysis of her original cohort, but her change of preference will be noted.

### Concomitant care and follow-up visits

Treatment of the patients will be conducted by standard of care regardless of trial participation. Routine clinical assessment will be performed in accordance with the standard of care. No extra trial-specific clinical follow-up assessments will be performed.

### Randomised patients and randomisation processes

Participants that accept randomisation will be randomised in a 1:1 ratio, using simple randomisation, with equal probability, to either autologous or implant-based reconstruction (figure 3). The mechanism of implementing the allocation sequence is sealed envelope. Allocation sequence will be ensured as the sequence will be concealed for participants, surgeons and research staff until the participant has been included in the randomised arm, which takes place after all inclusion and exclusion criteria have been checked, the PEGASUS intervention performed, and baseline clinical evaluation completed at the appointment with the plastic surgeon. All patients giving consent to participate in the randomised cohort that fulfils inclusion criteria will be randomised. Randomisation will be conducted without any influence from the surgeons/researchers. The intervention nature does not allow blinding.

In the patient preference cohort, patients will be included until the minimum targeted sample size has been reached, that is, until the minimum number of participants has selected the least popular alternative (figure 3).

### Outcomes

#### Primary outcome: satisfaction with the breast/s and breast-specific quality of life

BREAST-Q reconstruction module (version 1) is a validated disease-specific patient-reported instrument that measures outcomes after breast reconstruction, breast-related quality of life and patient satisfaction.^25^,^27^ The following domains will be analysed: satisfaction with breast/s, satisfaction with outcome, psychosocial well-being chest, sexual well-being, physical well-being chest and satisfaction with information. The patient rates all items in the domains on 3-point, 4-point and 5-point Likert scales. A raw score that is converted to a score of 0–100, is calculated for each domain. A higher score indicates greater satisfaction or a better quality of life. Normative data have been described for a Swedish population^36^ and it will be used for reference values. A further validation of the Swedish version is ongoing in our department.

#### Secondary outcomes

All adverse events are classified according to the Clavien-Dindo Classification (CDC) of surgical complications and Comprehensive Complication Index (CCI) scores,^37^ as well as specific complications. CDC and CCI are currently being validated for breast reconstruction in our department. All participating surgeons will be given a list of study-specific definitions of *complications and corrections/revisions. Satisfaction with the donor-site* will be measured with BREAST-Q donor site domains, expectations with BREAST-Q expectations domain,^38 39^ *symptoms of depression and anxiety* with Hospital Anxiety and Depression Scale (HADS),^40 41^ *body image* with the Appearance Schemas Inventory-revised (ASI-R)^42^and the Multidimensional Body-Self Relation Questionnaire (MBSRQ),^43^ *generic quality of life* with EuroQoL-5 dimensions (EQ-5D-3L),^44^ and the *patient’s goals with the reconstruction* will be documented using PEGASUS.^20^

The BREAST-Q donor site module has two domains (satisfaction with abdomen and physical well-being: abdomen) and the expectations module has six domains (support from medical staff, pain: postop, coping, appearance: clothes, sensation: breasts, and function: abdomen) and they are scored as described under ‘primary outcomes’. HADS^41^ measures symptoms of anxiety and depression in somatically ill patients on a Likert scale. For both domains, scores of less than 7 indicate non-cases, whereas scores of 8–10 indicate possible cases and scores of >10 indicate probable cases.^40 45 46^ The ASI-R measures body image investment, how important the individual believes their physical appearance is for her/his own self-worth. It is measured on Likert scales and has two domains: self-evaluative salience and motivational salience. The scores for the two domains are calculated as the mean of the items for each subscale. The total ASI-R score is the mean of all 20 items. A higher score indicates greater body image investment.^47^ The MBSRQ measures appearance-related aspects of body image on Likert scales and has four domains: appearance evaluation, appearance orientation, body areas satisfaction and overweight preoccupation.^43^ EQ-5D-3L has five dimensions: mobility, self-care, usual activities, pain/discomfort and anxiety/depression. The patient rates his/her health on a three-level Likert scale and a score is calculated, where 1 indicates ‘perfect health’ and 0 ‘death’. EQ-5D-3L The instrument also comprises a visual analogue scale where the patient marks his/her current health state, from 0 (‘worst imaginable’) to 100 (‘best imaginable’).^44 48^ The PEGASUS instrument is described under ‘Uniform preoperative counselling’.

### Study within a trial

The RPPT design will be assessed quantitatively using a SWAT.^22^ This considers the rate of patients fulfilling the criteria and agreeing to participate in the study, the time it takes to recruit the target number in each group, the rate of patients who accept randomisation and differences between the randomisation and preference cohort regarding demographic factors as well as preoperative satisfaction with breasts, expectations, body image, symptoms of depression and anxiety, and generic quality of life. To obtain insights into attitudes towards and experiences of the study process, semistructured interviews will be conducted concerning issues such as how the process can be ameliorated to increase recruitment, retention and follow-up rates of questionnaires, and how the participation information leaflet should be improved to maximise recruitment. Trial participants, participating surgeons and research nurses will be interviewed. Embedded qualitative studies will be used to investigate the participants’ thoughts, attitudes and experiences regarding:

- *The choice of breast reconstruction and the choice of reconstructive method*. Participants will be recruited from the preference cohort. Longitudinal—the same participants will be interviewed at allocation and 12 months after the reconstruction.
- *What makes a participant very satisfied or very dissatisfied*. Participants will be recruited from both cohorts among women who scored high/low, compared with the mean, on BREAST-Q outcome and satisfaction with breast/s.
- *SWAT*^22^*: How the participant experienced the trial process, how it can be ameliorated to increase recruitment, retention and follow-up rates of questionnaires and how the participation information leaflet should be designed/written to maximise recruitment*. Different participants from both cohorts are interviewed at allocation, 3 and 12 months to explore if there are different themes at different time points.

**Figure 4:**
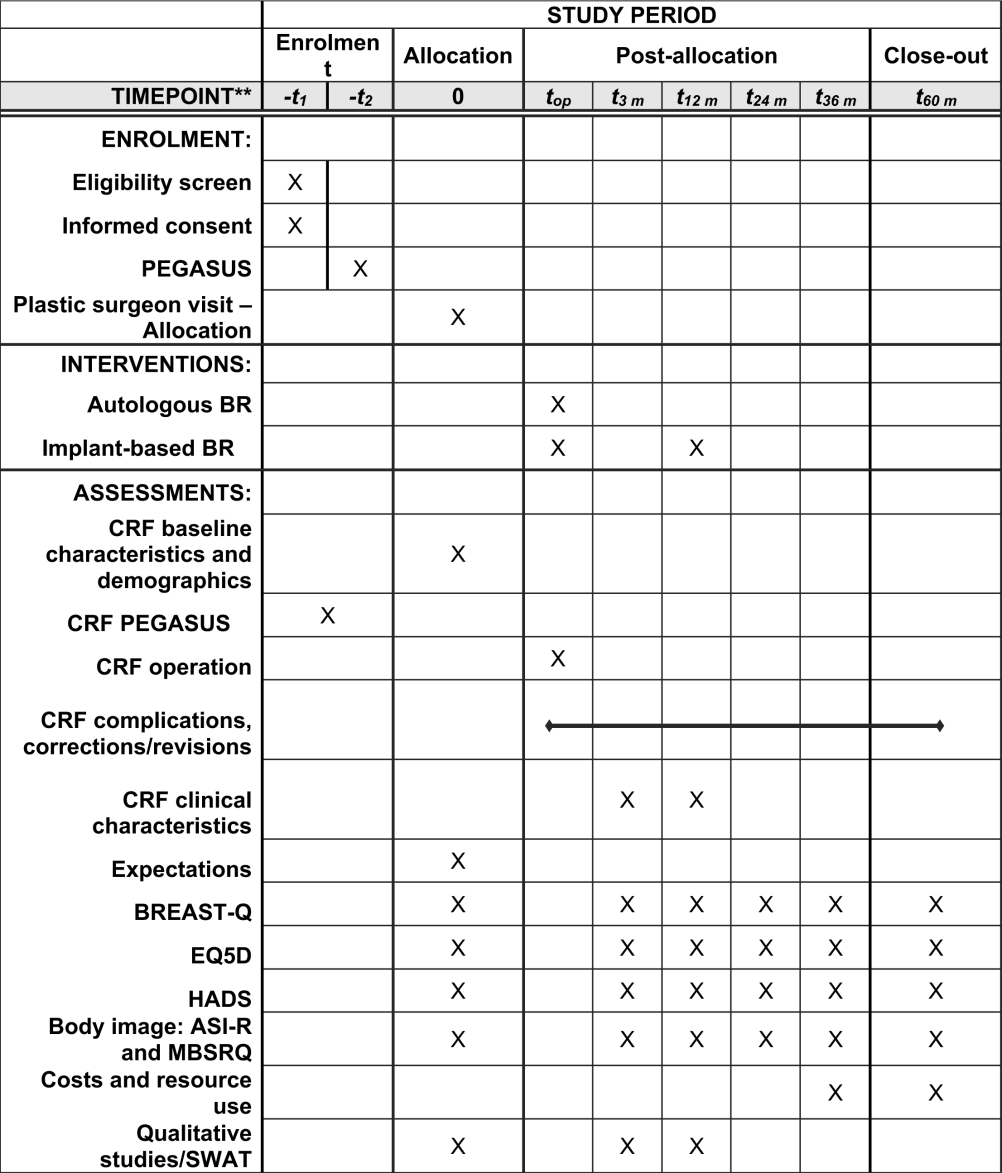
Trial flow chart for the participants. ASI-R, the Appearance Schemas Inventory-revised; BR, breast reconstruction; CRF, clinical report form; EQ5D, EuroQoL-5 dimensions; HADS, Hospital Anxiety and Depression Scale; MBRSQ, the Multidimensional Body-Self Relation Questionnaire; PEGASUS, Patients’ Expectations and Goals of reconstruction. Assisting Shared Understanding of Surgery; SWAT, study within a trial; t, timepoint.

Qualitative approaches and research paradigms will be chosen based on the type of question studied. Interview guides are given as online supplemental appendix 3. A purposive criterion-sampling technique will be used at the time points described in figure 4. Interviews will follow semistructured interview guides designed for each research question. Participants will be recruited until saturation has been achieved.

### Health economic analysis

A within-trial economic evaluation will be performed 36 months post allocation. The primary economic analysis will be a cost-effectiveness analysis presenting incremental cost-effectiveness ratios (ICERs). Effects will be expressed in terms of quality-adjusted life years, where the health-related quality of life will be assessed based on EQ-5D-3L using the UK Dolan tariff as well as a Swedish population-based tariff.^48 49^ A societal perspective will be adopted that includes healthcare costs and broader societal economic costs from sick leave based on the human capital approach. The healthcare costs will be based on inpatient, outpatient and primary care resource use and be collected from the controller of our departments (actual costs for the care given excluding any costs driven by the study protocols) and from Vega, the healthcare use database in Region Västra Götaland (VGR). Information on sick leave will be collected from the Swedish Social Insurance Agency (*Försäkringskassan* (https://www.forsakringskassan.se/english)) and information on the average income in different age groups from Statistics Sweden (*Statistikmyndigheten*, SCB (https://www.scb.se/en/)). ICERs and incremental net benefit (monetary/health) statistics will be assessed to compare the two interventions. The uncertainty will be assessed by non-parametric bootstrapping and visualised by means of cost-effectiveness planes and cost-effectiveness acceptability curves.

### Data collection and participant timeline

The participant timeline for the trial is shown in figure 4.

### Statistical methods

A detailed statistical analysis plan will be drafted early in the trial and finalised before primary outcome analysis. All analysis will be performed on an intention-to-treat basis and per-protocol analysis as sensitivity analysis. Descriptive data will be given as appropriate according to type of data and if it is skewed or not. This will also form the basis for statistical tests chosen to compare groups. Sensitivity analysis will be done were missing data in predictors will be handled by multiple imputation methods.^50^ Regression analysis preceded by collinearity check of potential predictors and performed to allow for correction for possible confounders. Residuals for each regression analysis will be checked for the assumptions of normal distribution and constant deviation along the predicted values. Subgroup analyses will be performed for the randomised and the preference cohort and for timing of reconstruction (immediate/delayed reconstruction) and for patients who unexpectedly will require radiotherapy. The statistical analysis will adhere to the Setting Intenartional Standards in Analysing Patient-Reported Outcomes and Quality of Life Endpoints in Cancer Clinical Trials (SISOQOL) framework.^51^ All tests will be two tailed and a p value of ≤0.05 will be considered to indicate a statistical significance.

### Adherence

Trial participants will be given their scheduled follow-up appointment at hospital discharge, with the following scheduled by post. A reminder text message will be sent prior to the appointments to improve adherence. Patient-reported outcome measure instruments will be given to the patient when they are allocated to treatment and then sent by mail at the subsequent timepoints. Two reminders will be sent to the patients if a questionnaire reply is not received by the hospital. Questionnaires will be given to the participants at allocation and then sent by mail, with up to two reminders, at the remaining timepoints, with reminders to ensure continued participation.

### Retention

Any trial participant lost to follow-up will be contacted to complete the 3, 12, 36 and 60 months follow-up. The trialists will make every reasonable effort to follow participants for the entire study period. If available, a reason for withdrawal will be documented.

### Data management, confidentiality and access to data

Arrangements for data handling and processing of personal data are detailed in the data management plan (DMP). All data will be handled according to the *General Data Protection Regulation* 2016/679 (GDPR), confidentiality offered by Swedish law (Offentlighets- och sekretesslagen (2009:400)), the ethical permit, and guidelines of the Swedish Authority for Privacy Protection (Integritetsskyddsmyndigheten, IMY (https://www.imy.se/en/)) and of the data controller and sponsor VGR. A data protection officer has been appointed by VGR. The lawfulness of data processing is a necessity for the performance of a task carried out in the public interest or in the exercise of official authority vested in the controller (art 6, GDPR). Data and metadata are collected and stored on paper within secure locations, in a locked cabinet approved for storage of class 3 and 4 information. Working files and continuous documentation are collected using VGR-licensed computer software on password-protected computers maintained by VGR. Storage and backup are performed in accordance with the guidelines of VGR. The filing system is registered in accordance with the guidelines of VGR. Data provenance is documented through codes (pseudonymised). Coding lists are stored and sealed according to the local routine of the Department of Plastic Surgery. All documentation and data will be archived for 25 years in the VGR repository according to VGR guidelines. Clinical trial participant-level data (IPD) will not be shared due to confidentiality. To ensure data quality during the life of the study, it is monitored as described in the DMP.

### Monitoring

VGR and the University of Gothenburg will undertake the role of sponsors in accordance with local guidelines. VGR will act as data controller. Delegated responsibilities will be assigned to the principal investigator, participating researchers and research nurses. The full coapplication team and clinical staff responsible for the day-to-day management of the trial will form the trial management group, which is responsible for monitoring recruitment and retention. No separate data monitoring committee is planned for this single-centre study.

### Safety and harms

The trial interventions are identical to the usual clinical practice. The only difference is that non-irradiated patients are offered autologous reconstruction, an option usually reserved for irradiated patients. Autologous breast reconstruction has been performed in our department since 1979^52^ and we currently perform around 120 per year in irradiated patients. Similarly, questionnaires can be sent to patients in the usual clinical practice to monitor their progress. Therefore, the risks of participating in the trial are considered similar to those of usual clinical practice. Adverse events are defined as any undesirable event occurring to the patient during the study period. All possible adverse events will be documented on clinical report forms (CRFs) and in the medical charts according to standard procedures for clinical trials and good clinical practice. All implants will be registered in the Swedish breast implant registry (https://brimp.registercentrum.se).

The Swedish healthcare service covers all the healthcare needs of the inhabitants and, thus, of the trial participants during and after the trial. Patients enrolled in the study are covered by the standard insurance and indemnity of the Swedish public healthcare service (*Löf regionernas ömsesidiga försäkringsbolag* (https://lof.se/language/engelska-english)).

## Ethics and dissemination

The study has been approved by the Swedish Ethical Review Authority (https://etikprovningsmyndigheten.se/en/) (2023-04754-01). Any protocol amendments or ancillary studies will be vetted by the Swedish Ethical Review Authority.

Results will be published in peer-reviewed scientific journals and presented at peer-reviewed scientific meetings. Researchers and trialists that have made a substantive contribution in accordance with the Vancouver recommendations for authorships and fulfil the criteria and requirements of the International Committee of Medical Journal Editors (https://www.icmje.org/recommendations/browse/roles-and-responsibilities/defining-the-role-of-authors-and-contributors.html) will be listed as authors for the publications. Those who do not fulfil the criteria will not be granted authorship. The Contributor Roles Taxonomy (https://credit.niso.org) will be used to declare the authors’ roles for every manuscript. Professional medical writers will not be employed.

The datasets used and analysed during the studies will not be published in a public depository but will be available from the corresponding author on reasonable request, ethical permission and compliance with GDPR and Swedish law (cf. ‘Data management, confidentiality, and access to data’).

### Consent or assent

Participants that are eligible for inclusion will be invited and given written and oral standardised information and have the chance to ask any questions about the trial. Patients who consent verbally to participation will be asked to sign a written consent form before allocation (online supplemental appendix 4). A similar, but separate, consent process will be performed for participants asked for inclusion in the qualitative studies and in the SWAT.^22^ Qualitative studies will be performed at different time points for different research questions and the participants will be informed about the studies and asked for consent at these timepoints. The participant will be contacted by phone and informed about the study by the researcher who performs the interviews and then sent written information about the study. A week later, the participant will receive a new phone call asking for consent and booking of date for interview. The quantitative SWAT analyses are included in the basic consent for the study. Participants are free to withdraw at any time and for any reason, without consequence. Participants that withdraw from follow-up questionnaires may continue to consent for data collection from CRFs and clinical records. Data collected prior to withdrawal may be retained and used in the analyses if the participants consent to it.

### Declaration of interests

The principal investigator and participating researchers have no financial or other competing interests to declare.

### Patient and public involvement

Official representatives of the Breast Cancer Association have participated in the planning of the study and are coauthors of this protocol (CL, AU and KS). The group will be collaborators in the study, throughout its course, and in the analysis of the results and writing of the manuscripts.

A qualitative study embedded in the first GoBreast study (*manuscript under writing*) has demonstrated that the patients scoring low on satisfaction with breast on BREAST-Q often attribute their low satisfaction to a feeling of not being involved in the decision around breast reconstruction and that they have not been allowed to make their own informed decisions. The lack of patient involvement and adequate preoperative information was also clearly seen in a report released by The Swedish Breast Cancer Association.^4^ This led our department to implement use of the PEGASUS instrument.^30^ The instrument has been incorporated in the protocol and will form a basis for a patient-centred dialogue and emphasise the possibility to make a choice according to preferences, if the patient has any.

The primary and secondary outcomes of the study are based on the published core outcomes set for breast reconstruction, which has been developed by patients and professionals. All outcomes considered important by patients in that previous study have been incorporated into our study.

### Funding

The study is funded by the Swedish Cancer Society (grant number 23 3240 S).

## Discussion

GoBreast II will mark the first use of an RPPT design to evaluate breast reconstruction methods. The trial builds on GoBreast, which randomised irradiated patients to a DIEP-flap or latissimus dorsi-flap with an implant and non-irradiated patients to a thoracodorsal flap and implant or implant-based breast reconstruction in two stages.^18 19^ The study illustrated the described difficulties with conducting an RCT in breast reconstruction.

### Considerations regarding the RPPT design and methodological significance

Development of the RPPT design has the potential to become a standard when preference sensitive treatment options are compared in all disciplines and types of cancer clinical therapy studies. If successful in this trial, we plan to use it to study fundamental questions like immediate versus delayed breast reconstruction, for which there is very little scientific evidence and thus far no ethically acceptable designs. Through SWATs, the design will also give us information about how many women prefer the two options and how many women accept randomisation, which will help in planning resource usage in breast reconstruction as well as the design of future studies.

### Considerations regarding the included population and equipoise

In the present study, only non-irradiated patients will be included. Non-irradiated patients are the only group in which there is a theoretical equipoise in the reconstructive community, as it is well known that implants and other foreign materials and radiotherapy do not marry well.^53^ Therefore, irradiated patients are not included in the present study.

In recent years, the choice of breast reconstruction method might have been increasingly affected by professional conflicts and the individual surgeon’s competence rather than the patient’s preferences and suitability for different methods.^54^ The main conflict is which speciality should perform the reconstruction. In some units, general surgeons specialised in breast surgery, with skills mainly in implant-based breast reconstruction, have assumed responsibility for implant-based breast reconstruction and the primary discussion regarding breast reconstruction. Only patients actively requesting autologous breast reconstruction are referred to a plastic surgeon, who usually has a broader competence in reconstructive methods. The conflict might also have led to many plastic surgeons actively promoting autologous breast reconstruction as this is their best possibility to have patients referred. Hence, the information about different options the patient receives might be biased by the competence and interests of the surgeon,^55^,^57^ limiting a shared decision-making process which is essential in preference-sensitive interventions.^58^,^62^ The lack of standardised information and access to different options is also reflected in a report published by the Swedish Breast Cancer Association.^4^ Moreover, commercial factors, such as the marketing of implants to surgeons and pressure by health insurance companies for patients to undergo implant-based rather than autologous reconstruction, could have an impact on the choice of reconstructive method. These are not factors in Sweden, as the surgeons’ pay is independent of the method used, and the healthcare system is a publicly funded welfare-type healthcare system. In the interest of the patients, the results of an RPPT comparing implant-based and autologous breast reconstruction in non-irradiated could create a basis for a standard on what information patients should receive when facing choices of reconstructive breast surgery and ultimately equal access to care.

### Considerations regarding the choice of outcomes

A core outcome set has been developed for breast reconstruction.^21^ Core items for patients as well as professionals include major complications, unplanned surgery for any reason, donor site problems/morbidity, normality, quality of life and women’s cosmetic satisfaction.^21^ In addition, professionals consider implant-related complications and flap-related complications to be core items, and patients believe self-esteem, emotional well-being and physical well-being are important outcomes.^21^ The core outcomes set forms the basis for the outcomes included in the present study. However, the outcome set does not give any recommendations regarding how the different outcomes should be measured.

Our department conducts a project (*ValPlast*) (ClinicalTrials.gov identifier NCT0523389) where patient-reported outcome instruments and complication classifications are validated for use in Swedish for breast reconstruction and where Swedish norms are created and projects on how complications and other factors affect the outcomes in breast reconstruction^63 64^ (ClinicalTrials.gov identifier NCT04714463). The results form the basis for the choices of instrument in the present study.

### Considerations regarding the health economic analysis

A systematic review on health economics in breast reconstruction has demonstrated that there is no high-level evidence, regarding cost-effectiveness, to support recommendations and decisions in breast reconstruction.^8^ The review^8^ identified several methodological issues, such as a lack of a societal perspective, usage of standardised and validated methods to evaluate benefits, and modelling approaches not compatible with the reconstructive reality. The identified methodological weaknesses have formed the basis of the design of the present study.

### Risks with the study

The most prominent operational risk in the project is that autologous reconstruction inherently requires more healthcare resources than implant-based reconstruction. Currently, one autologous reconstruction can be performed a day per operation theatre, while three implant-based reconstructions can be performed. An autologous reconstruction also requires surgeons with skills in microsurgery. Through training fellowships, we have invested in the necessary specialist competence and currently have five surgeons performing autologous reconstruction, allowing for a considerable expansion. We are prepared to reach the target in the randomised groups if there should be an increase in autologous reconstructions. Despite performing about 350–400 reconstructions per year in our department, conceptual risks include difficulties in recruiting participants, especially to the randomised arm, and a low adherence and retention. We expect that it will be easier to recruit when participants know that they will be treated according to their preferences.

### Significance

The study could provide evidence of which reconstruction method is superior to increase women’s breast-related quality of life and is the most cost-effective for society. This can facilitate the making of guidelines for breast reconstruction in healthcare. Evidence of the cost-effectiveness of alternative treatments can also be used to influence how politicians allocate budget resources so that more women have access to the best methods for breast reconstruction.

Knowledge about women’s experiences of choices and the reconstructive process can improve shared decision-making in breast reconstruction and serve as a basis for standardising information and the breast cancer processes and multidisciplinary collaboration regarding reconstruction. The lack of standardised information and access to different options has been illustrated in a report published by the Swedish breast cancer association (https://brostcancerforbundet.se/wt/documents/918/Bröstcancerrapport2021final3.pdf)https://brostcancerforbundet.se/wt/documents/918/Br. Our qualitative studies also have the potential to identify knowledge gaps that should be explored in future studies.

## supplementary material

10.1136/bmjopen-2024-084025 online supplemental appendix 1

10.1136/bmjopen-2024-084025 online supplemental appendix 2

10.1136/bmjopen-2024-084025 online supplemental appendix 3

10.1136/bmjopen-2024-084025 online supplemental appendix 4
