# Supplementary material for: Gothenburg Breast reconstruction (GoBreast) II protocol: a Swedish partially randomised patient preference, superiority trial comparing autologous and implant-based breast reconstruction
Source: BMJ Open. 2024 Jul 17;14(7):e084025. doi: 10.1136/bmjopen-2024-084025 (PMC11256070; doi:10.1136/bmjopen-2024-084025)
Supplement: online supplemental appendix 3 [file bmjopen-14-7-s003.pdf]

GoBreast II: Interview guides

The choice to undergo reconstruction and the choice of method

The overall aim of the study will be to investigate how women who have had breast reconstruction experience the process.

• What do you think today about your reconstruction and your choice?
• How would you describe your relationship with your breasts before your breast cancer/risk-reducing mastectomy?
• What information about different surgical methods to remove the tumour did you get?
• What recommendations did you receive regarding surgical treatment?
• What do you think about the choices and the information you received afterwards?
• What would you have liked to know before the operation?
• What were your expectations for/goals with the breast reconstruction?
• Did you think it was easy to decide whether you wanted breast reconstruction or not?
• Who did you discuss breast reconstruction with? (patient association, relative, friend, etc.)
• Did you feel any pressure from anyone else to have breast reconstruction?
• How did you experience the information you received from the healthcare provider about breast reconstruction?
• Did you feel involved in the decisions that were made regarding breast reconstruction?
• Do you regret any of the decisions you made (that were made)? Would you have chosen differently if you had had the experiences you have today?
• Would you have liked some other form of help to make decisions about breast reconstruction?

High degree of satisfaction or strong dissatisfaction with the breast reconstruction?

The overall aim of the study will be to investigate how women describe what it is that makes them feel very satisfied or very dissatisfied with their breast reconstruction.

• The process up to reconstruction.
• The own choice. Did you feel involved in the decisions that were made regarding breast reconstruction?
• Would you have liked some other form of help to make decisions about breast reconstruction?
• How did you perceive the information you received from the health care system regarding breast reconstruction?
• Did you receive any recommendations for reconstruction?
• How would you describe your relationship with your breasts before your breast cancer/risk-reducing mastectomy?
• Did you feel any pressure from anyone else/external pressure to have breast reconstruction?
• What went well/as you imagined? What went bad/not as you expected?

• What were your expectations before the breast reconstruction/goals with the reconstruction?
• Do you regret any of the decisions you made (that were made)? Would you have chosen differently if you had had the experiences you have today?
• Do you feel that the health care system should have done/handled something differently?
• Did you discuss possibilities and limitations and what is possible to achieve with your surgeon?
• Has your surgeon recommended any corrections?
• How would you have like it to be (process, choice, outcome)?
• Do you feel that you have changed as a person? Do others see/treat you differently because of what happened to your breasts?

SWAT

• How did you experience the inclusion in the study?
• What did you think of the choice between choosing yourself or being randomized to a method?
• Do you have any suggestions regarding how the process can be improved? How do you think we can increase participation and increase questionnaire response rates?
• What do you think about the research person information?
• What did you like most/least about the study?
