# Supplementary material for: Gothenburg Breast reconstruction (GoBreast) II protocol: a Swedish partially randomised patient preference, superiority trial comparing autologous and implant-based breast reconstruction
Source: BMJ Open. 2024 Jul 17;14(7):e084025. doi: 10.1136/bmjopen-2024-084025 (PMC11256070; doi:10.1136/bmjopen-2024-084025)
Supplement: online supplemental appendix 4 [file bmjopen-14-7-s004.pdf]

Information till forskningspersoner

Vi vill fråga dig om du vill delta i ett forskningsprojekt. I det här dokumentet får du information om projektet och om vad det innebär att delta.

Vad är det för ett projekt och varför vill ni att jag ska delta?

I detta forskningsprojekt önskar vi jämföra två metoder för bröstrekonstruktion, återskapande av bröst, vid cancer/hög risk för cancer: kroppsegen och implantatbaserad teknik. Du tillfrågas då ditt/dina bröst planeras opereras bort/har opererats bort.

Enligt nuvarande riktlinjer erbjuds kroppsegen rekonstruktion främst till kvinnor som har fått strålbehandling då kroppsegen bröstrekonstruktion är en resurskrävande operation och det är de som fått strålbehandling som har störst behov av tillförsel av icke-strålad vävnad till området. Kvinnor som inte har fått strålbehandling erbjuds enligt riktlinjerna främst implantatbaserad rekonstruktion.

I detta projekt önskar vi jämföra om kvinnor som inte har fått strålbehandling blir mest nöjda med kroppsegen eller implantatbaserad rekonstruktion, s.k. patientrapporterat utfall. De två metoderna kommer också att jämföras med avseende på komplikationer och kostnadseffektivitet på lång sikt.

Den högsta vetenskapliga bevisnivå anser man att man får då man gör en s.k. randomiserad studie där studiedeltagaren lottas till ett av de två behandlingsalternativen och studien kommer därför ha denna design. Eftersom bröstrekonstruktion är en s.k. preferenssensitiv åtgärd där många kvinnor har starka önskemål om att bli opererad med en viss metod kommer det vara möjligt att avstå lottning och själv välja metod om du önskar det. Datan från denna studie kommer även att användas för att studera den vetenskapliga metoden att låta vissa deltagare välja och lotta andra deltagare samt om det finns skillnader mellan de två grupperna, t.ex. vad gäller demografi eller mål med rekonstruktion.

Forskningshuvudman för projektet är Verksamhet plastikkirurgi, Sahlgrenska universitetssjukhuset, Västra Götalandsregionen och Göteborgs universitet. Med forskningshuvudman menas den organisation som är ansvarig för projektet. Ansökan är godkänd av Etikprövningsmyndigheten, diarienummer för prövningen hos Etikprövningsmyndigheten är 2023-04754-01

Hur går projektet till?

Ett deltagande innebär att du antingen lottas till kroppsegen eller implantatbaserad rekonstruktion (lottad grupp) eller själv välja vilken metod du vill bli opererad med, om du har starka önskemål kring metod (preferensgrupp). Innan du lottas/väljer metod kommer du

att få träffa först sjuksköterska eller psykolog vid ett besök för att diskutera dina mål med en rekonstruktion och sedan plastikkirurg vid ett andra besök för att diskutera olika tekniker. Dina mål med rekonstruktionen kommer att dokumenteras enligt en modell som heter PEGASUS.

Innan och efter operationen (3, 12, 24, 36 och 60 månader efter) kommer du att få svara på enkäter angående din nöjdhet med operationen, resultatet och vården samt hur du mår i övrigt. Det tar ungefär 20 minuter att fylla i enkäterna vid vart tillfälle. ***Det är mycket viktigt att du kan tänka dig ta dig tiden att fylla i enkäterna vid alla tillfällen, för att vi ska kunna få ett pålitligt resultat.*** Förutom lottning/val av teknik kommer vården att vara exakt den samma som om du inte hade deltagit i studien. Studien innebär inga extra besök på sjukhus efter operationen, än de som du skulle ha gått på om du inte deltar i studien.

Möjliga följder och risker med att delta i projektet

Om du deltar i projektet kommer du att få samma behandling som om du inte hade deltagit i projektet, med undantag att du som icke-strålad kan komma att opereras med kroppsegen i stället för implantatbaserad rekonstruktion. Både kroppsegen och implantatbaserad bröstrekonstruktion är rutinbehandling som utförs varje vecka på sjukhuset och som Sahlgrenska har utfört sedan 1970-talet.

Då projektet innebär att du svarar på enkäter innan och vid 5 tillfällen efter operationen innebär det att du kommer att bidra med din tid till forskningsprojektet, utan att få ekonomisk ersättning för detta. Att svara på enkäterna skulle också kunna innebära att du påminns om tidigare cancerbehandling/cancerriskreducerande behandling, vilket kan vara känslomässigt obehagligt. Om du önskar ytterligare hjälp att bearbeta dina upplevelser är du välkommen att höra av dig till plastikkirurgen, så lotsar vi dig rätt i sjukvårdssystemet.

Vad händer med mina uppgifter?

Projektet kommer att samla in och registrera information om dig.

Enkäterna kommer att kodas och förvaras inlåsta. De kommer att arkiveras i 25 år. Kodlistan kommer att förvaras inlåst och separerad från enkäterna. Du kommer antingen få enkäterna i samband med mottagningsbesök eller hemskickade till dig med ett frankerat svarskuvert.

För att kunna tolka enkätsvaren kommer vi att samla in data från din journal. Detta inkluderar detaljer kring tidigare sjukdomar, den/de operation/er du genomgått samt om orsakerna till att du opererats, dina mål med rekonstruktionen, ditt hälsotillstånd vid operationstillfället samt information kring vårdförloppet (t.ex. antal besök på mottagningen och recept på smärtstillande).

För att kunna undersöka kostnadseffektivitet kommer vi också inhämta data från Västra Götalands databas kring annan vård som du har haft behov av under perioden, t.ex. från din vårdcentral eller från andra kliniker, samt från Försäkringskassan angående din sjukskrivningslängd och ytterligare behov av sjukskrivning.

Informationen kommer bara vara tillgänglig för forskningspersonerna och förvaras i kodad form, så de inte kan härledas till dig. Uppgifterna skyddas som journalhandling

Den rättsliga grunden för databehandlingen enligt EU:s dataskyddsförordning är att den är nödvändig för att utföra en uppgift av allmänt intresse och som ett led i Västra Götalands myndighetsutövning, dvs. uppgiften att utföra forskning (artikel 6 e).

Dina svar och dina resultat kommer att behandlas så att inte obehöriga kan ta del av dem.
Ansvarig för dina personuppgifter är Verksamhet plastikkirurgi, Sahlgrenska universitetssjukhuset, Västra Götalandsregionen. Enligt EU:s dataskyddsförordning har du rätt att kostnadsfritt få ta del av de uppgifter om dig som hanteras i projektet, och vid behov få eventuella fel rättade. Du kan också begära att uppgifter om dig raderas samt att behandlingen av dina personuppgifter begränsas. Rätten till radering och till begränsning av behandling av personuppgifter gäller dock inte när uppgifterna är nödvändiga för den aktuella forskningen. Om du vill ta del av uppgifterna ska du kontakta huvudansvarig forskare Emma Hansson, Gröna Stråket 8, 413 45 Göteborg. Tel. 031-3423700. Dataskyddsombud nås på sahlgrenska.universitetssjukhuset.dso@vgregion.se . Om du är missnöjd med hur dina personuppgifter behandlas har du rätt att ge in klagomål till Integritetsskyddsmyndigheten, som är tillsynsmyndighet.

Hur får jag information om resultatet av projektet?

Resultatet kommer att presenteras på gruppnivå i vetenskapliga artiklar. Om du önskar kopia på den data som finns kring dig kan du begära kopia av dina enkätsvar från projektansvariga (kontaktuppgifter i slutet på denna information).

Försäkring och ersättning

Sedvanlig patientskadeförsäkring gäller. Ingen ersättning utgår för deltagande i projektet.

Deltagandet är frivilligt

Ditt deltagande är frivilligt och du kan när som helst välja att avbryta deltagandet. Om du väljer att inte delta eller vill avbryta ditt deltagande behöver du inte uppge varför, och det kommer inte heller att påverka din framtida vård eller behandling.

Om du vill avbryta ditt deltagande ska du kontakta den ansvariga för projektet (se nedan).

Ansvariga för projektet

Ansvariga för projektet är FOU-sjuksköterska Susanne Meyer, susanne.meyer@vgregion.se Lektor Anna Paganini, specialistsjuksköterska, med dr. anna.paganini@vgregion.se, prof. Emma Hansson, öl, emma.em.hansson@vgregion.se Alla tre nås på: Verksamhet plastikkirurgi, Sahlgrenska universitetssjukhuset, Gröna Stråket 8, 413 45 Göteborg. Tel. 031-3423700 .

Samtycke till att delta i projektet

Jag har fått muntlig och/eller skriftlig information om studien och har haft möjlighet att ställa frågor. Jag får behålla den skriftliga informationen.

- ☐ Jag samtycker till att delta i projektet *Kroppsegen jämfört med implantatbaserad bröstrekonstruktion - en delvis lottad patientpreferensstudie med 5 års uppföljning (GoBreast II)*
  Randomiserad kohort och preferenskohort

Plats och datum	Underskrift
	Namnförtydligande

Information till forskningspersoner

Vi vill fråga dig om du vill delta i ett forskningsprojekt. I det här dokumentet får du information om projektet och om vad det innebär att delta.

Vad är det för ett projekt och varför vill ni att jag ska delta?

I detta forskningsprojekt önskar vi undersöka dina upplevelser och erfarenheter kring att delta i GoBreast II, där du antingen har lottats eller valt mellan två metoder för bröstrekonstruktion. Denna studiedesign har tidigare aldrig använts för att studera olika s.k. preferenssensitiva åtgärder inom bröstrekonstruktion och syftet med denna del av projektet är att förfina metodologin inför framtida studier av preferenssensitiva åtgärder inom bröstcancervården i stort. Du tillfrågas om deltagande då du deltar i studien GoBreast II.

Forskningshuvudman för projektet är Västra Götalandsregionen. Med forskningshuvudman menas den organisation som är ansvarig för projektet. Ansökan är godkänd av Etikprövningsmyndigheten, diarienummer för prövningen hos Etikprövningsmyndigheten är 2023-04754-01

Hur går projektet till?

Ett deltagande innebär att du kommer att kallas för en intervju med frågor kring din upplevelse av att delta i GoBreast II samt vad du tycker bör ändras/kan förbättras. Intervjun kommer att utföras av en psykolog/psykologstudent eller en sjuksköterska eller en läkare.

Intervjun sker vid ett tillfälle och tar cirka 2 timmar och genomförs där du själv önskar (sjukhuset/hemma hos dig/på din arbetsplats) eller online (Zoom eller Teams).

Studiedeltagande innebär inga andra åtaganden från din sida och kommer inte påverka den vård eller det bemötande du får inom sjukvården

Möjliga följder och risker med att delta i projektet

Deltagande i projektet i studien skulle kunna leda till att gamla känslor kring bröstcancer och dess behandling väcks till liv. I fall det visar sig att du behöver ytterligare hjälp att bearbeta dina upplevelser kommer de forskningsansvariga att ombesörja att du remitteras till rätt vårdinstans för att få sådan hjälp.

Vad händer med mina uppgifter?

Projektet kommer att samla in och registrera information om dig.

Intervjuerna kommer att spelas in och sedan skrivas ut (transkriberas) anonymt. Texterna kommer att förvars kodade och inlåsta. Kodlistan kommer att förvaras inlåst och separerad

från utskrift från intervjuer. Texterna och inspelningarna kommer att arkiveras i 25 år. Informationen kommer bara vara tillgänglig för forskningspersonerna och förvaras i kodad form, så den inte kan härledas till dig. Uppgifterna skyddas som journalhandling. För att kunna tolka svaren kommer vi även att använda sedan data kring din behandling den/de operation/er du genomgått samt om orsakerna till att du opererats, ditt hälsotillstånd (t.ex. tobaksanvändning, vikt och längd) och ålder vid operationstillfället, som tidigare samlats in inom ramen för GoBreast II.

Dina svar och dina resultat kommer att behandlas så att inte obehöriga kan ta del av dem.

Ansvarig för dina personuppgifter (personuppgiftsansvarig) är Västra Götalandsregionen. Enligt EU:s dataskyddsförordning har du rätt att kostnadsfritt få ta del av de uppgifter om dig som hanteras i projektet, och vid behov få eventuella fel rättade. Du kan också begära att uppgifter om dig raderas samt att behandlingen av dina personuppgifter begränsas. Rätten till radering och till begränsning av behandling av personuppgifter gäller dock inte när uppgifterna är nödvändiga för den aktuella forskningen. Om du vill ta del av uppgifterna ska du kontakta huvudansvarig forskare Emma Hansson, Gröna Stråket 8, 413 45 Göteborg. Tel. 031-3423700. Dataskyddsombud nås på sahlgrenska.universitetssjukhuset.dso@vgregion.se . Om du är missnöjd med hur dina personuppgifter behandlas har du rätt att ge in klagomål till Integritetsskyddsmyndigheten, som är tillsynsmyndighet.

Hur får jag information om resultatet av projektet?

Resultatet kommer att presenteras på gruppnivå i vetenskapliga artiklar. Om du önskar kopia på din utskriva intervju kontaktar du forskningssjuksköterskan (kontaktuppgifter sist i detta dokument).

Försäkring och ersättning

Sedvanlig patientskadeförsäkring gäller. Ingen ersättning utgår för deltagande i projektet.

Deltagandet är frivilligt

Ditt deltagande är frivilligt och du kan när som helst välja att avbryta deltagandet. Om du väljer att inte delta eller vill avbryta ditt deltagande behöver du inte uppge varför, och det kommer inte heller att påverka din framtida vård eller behandling.

Om du vill avbryta ditt deltagande ska du kontakta den ansvariga för projektet (se nedan).

Ansvariga för projektet

Ansvariga för projektet är FOU-sjuksköterska Susanne Meyer, susanne.meyer@vgregion.se Lektor Anna Paganini, specialistsjuksköterska, med dr. anna.paganini@vgregion.se, prof. Emma Hansson, öl, emma.em.hansson@vgregion.se Alla tre nås på: Verksamhet

plastikkirurgi, Sahlgrenska universitetssjukhuset, Gröna Stråket 8, 413 45 Göteborg. Tel. 031-3423700 .

Samtycke till att delta i projektet

Jag har fått muntlig och/eller skriftlig information om studien och har haft möjlighet att ställa frågor. Jag får behålla den skriftliga informationen.

☐ Jag samtycker till att delta i projektet *Kroppsegen jämfört med implantatbaserad bröstrekonstruktion - en delvis lottad patientpreferensstudie med 5 års uppföljning (GoBreast II)*
Intervju kring metodologin lotta/välja (SWAT

Plats och datum	Underskrift
	Namnförtydligande

Information till forskningspersoner

Vi vill fråga dig om du vill delta i ett forskningsprojekt. I det här dokumentet får du information om projektet och om vad det innebär att delta.

Vad är det för ett projekt och varför vill ni att jag ska delta?

I detta forskningsprojekt önskar vi undersöka dina upplevelser och erfarenheter kring att rekrytera, operera och ta hand om patienter i GoBreast II-studien. Denna studiedesign har tidigare aldrig använts för att studera olika s.k. preferenssensitiva åtgärder inom bröstrekonstruktion och syftet med denna del av projektet är att förfina metodologin inför framtida studier av preferenssensitiva åtgärder inom bröstcancervården i stort. Du tillfrågas om deltagande då du har rekryterat/opererat/tagit hand om patienter inom ramen för studien GoBreast II.

Forskningshuvudman för projektet är Västra Götalandsregionen. Med forskningshuvudman menas den organisation som är ansvarig för projektet. Ansökan är godkänd av Etikprövningsmyndigheten, diarienummer för prövningen hos Etikprövningsmyndigheten är 2023-04754-01.

Hur går projektet till?

Ett deltagande innebär att du kommer att kallas för en intervju med frågor kring din upplevelse av att delta i GoBreast II samt vad du tycker bör ändras/kan förbättras. Intervjun kommer att utföras av en psykolog/psykologstudent eller en sjuksköterska eller en läkare.

Intervjun sker vid ett tillfälle och tar cirka 2 timmar och genomförs på sjukhuset eller online (Zoom eller Teams).

Möjliga följder och risker med att delta i projektet

Deltagandet i projektet innebär inga risker för dig som sjukvårdspersonal.

Vad händer med mina uppgifter?

Projektet kommer att samla in och registrera information om dig.

Intervjuerna kommer att spelas in och sedan skrivas ut (transkriberas) anonymt. Texterna kommer att förvars kodade och inlåsta. Kodlistan kommer att förvaras inlåst och separerad från utskrift från intervjuer. Texterna och inspelningarna kommer att arkiveras i 25 år. Informationen kommer bara vara tillgänglig för forskningspersonerna och förvaras i kodad form, så den inte kan härledas till dig. Uppgifterna skyddas som journalhandling. För att

kunna tolka svaren kommer vi även att samla information kring vilken profession du tillhör samt vilken roll du haft i GoBreast II.

Dina svar och dina resultat kommer att behandlas så att inte obehöriga kan ta del av dem.

Ansvarig för dina personuppgifter (personuppgiftsansvarig) är Västra Götalandsregionen. Enligt EU:s dataskyddsförordning har du rätt att kostnadsfritt få ta del av de uppgifter om dig som hanteras i projektet, och vid behov få eventuella fel rättade. Du kan också begära att uppgifter om dig raderas samt att behandlingen av dina personuppgifter begränsas. Rätten till radering och till begränsning av behandling av personuppgifter gäller dock inte när uppgifterna är nödvändiga för den aktuella forskningen. Om du vill ta del av uppgifterna ska du kontakta huvudansvarig forskare Emma Hansson, Gröna Stråket 8, 413 45 Göteborg. Tel. 031-3423700. Dataskyddsombud nås på sahlgrenska.universitetssjukhuset.dso@vgregion.se . Om du är missnöjd med hur dina personuppgifter behandlas har du rätt att ge in klagomål till Integritetsskyddsmyndigheten, som är tillsynsmyndighet.

Hur får jag information om resultatet av projektet?

Resultatet kommer att presenteras på gruppnivå i vetenskapliga artiklar. Om du önskar kopia på din utskriva intervju kontaktar du forskningssjuksköterskan (kontaktuppgifter sist i detta dokument).

Försäkring och ersättning

Sedvanlig patientskadeförsäkring gäller. Ingen ersättning utgår för deltagande i projektet.

Deltagandet är frivilligt

Ditt deltagande är frivilligt och du kan när som helst välja att avbryta deltagandet. Om du väljer att inte delta eller vill avbryta ditt deltagande behöver du inte uppge varför.

Om du vill avbryta ditt deltagande ska du kontakta den ansvariga för projektet (se nedan).

Ansvariga för projektet

Ansvariga för projektet är FOU-sjuksköterska Susanne Meyer, susanne.meyer@vgregion.se Lektor Anna Paganini, specialistsjuksköterska, med dr. anna.paganini@vgregion.se, prof. Emma Hansson, öl, emma.em.hansson@vgregion.se Alla tre nås på: Verksamhet plastikkirurgi, Sahlgrenska universitetssjukhuset, Gröna Stråket 8, 413 45 Göteborg. Tel. 031-3423700 .

Samtycke till att delta i projektet

Jag har fått muntlig och/eller skriftlig information om studien och har haft möjlighet att ställa frågor. Jag får behålla den skriftliga informationen.

☐ Jag samtycker till att delta i projektet *Kroppsegen jämfört med implantatbaserad bröstrekonstruktion - en delvis lottad patientpreferensstudie med 5 års uppföljning (GoBreast II)*
Intervju kring metodologin lotta/välja personal (SWAT)

Plats och datum	Underskrift
	Namnförtydligande

Information till forskningspersoner

Vi vill fråga dig om du vill delta i ett forskningsprojekt. I det här dokumentet får du information om projektet och om vad det innebär att delta.

Vad är det för ett projekt och varför vill ni att jag ska delta?

I detta forskningsprojekt önskar vi undersöka vad det är som att man blir väldigt nöjd eller väldigt missnöjd med en bröstrekonstruktion. Syftet är förbättra omhändertagandet av framtida patienter som önskar bröstrekonstruktion. Du tillfrågas om deltagande då du deltar i studien GoBreast II och har poängsatt ditt resultat som väldigt bra/väldigt dåligt.

Forskningshuvudman för projektet är Västra Götalandsregionen . Med forskningshuvudman menas den organisation som är ansvarig för projektet. Ansökan är godkänd av Etikprövningsmyndigheten, diarienummer för prövningen hos Etikprövningsmyndigheten är 2023-04754-01

Hur går projektet till?

Ett deltagande innebär att du kommer att kallas för en intervju med frågor kring vad som gjort dig väldigt nöjd/väldigt missnöjd. Intervjun kommer att utföras av en psykolog/psykologstudent, en sjuksköterska eller en läkare.

Intervjun sker vid ett tillfälle och tar cirka 2 timmar och genomförs där du själv önskar (sjukhuset/hemma hos dig/på din arbetsplats) eller online (Zoom eller Teams).

Studiedeltagande innebär inga andra åtaganden från din sida och kommer inte påverka den vård eller det bemötande du får inom sjukvården

Möjliga följder och risker med att delta i projektet

Deltagande i projektet i studien skulle kunna leda till att gamla känslor kring bröstcancer och dess behandling väcks till liv. I fall det visar sig att du behöver ytterligare hjälp att bearbeta dina upplevelser kommer de forskningsansvariga att ombesörja att du remitteras till rätt vårdinstans för att få sådan hjälp.

Vad händer med mina uppgifter?

Projektet kommer att samla in och registrera information om dig.

Intervjuerna kommer att spelas in och sedan skrivas ut (transkriberas) anonymt. Texterna kommer att förvars kodade och inlåsta. Kodlistan kommer att förvaras inlåst och separerad från utskrift från intervjuer. Texterna och inspelningarna kommer att arkiveras i 25 år.

Informationen kommer bara vara tillgänglig för forskningspersonerna och förvaras i kodad form, så de inte kan härledas till dig. Uppgifterna skyddas som journalhandling. För att kunna tolka svaren kommer vi även att använda sedan data kring din behandling den/de operation/er du genomgått samt om orsakerna till att du opererats, ditt hälsotillstånd (t.ex. tobaksanvändning, vikt och längd), ålder vid operationstillfället och den lista över mål med rekonstruktion du kom fram till innan operationen (PEGASUS), som tidigare samlats in inom ramen för GoBreast II.

Dina svar och dina resultat kommer att behandlas så att inte obehöriga kan ta del av dem.

Ansvarig för dina personuppgifter (personuppgiftsansansvarig) är Västra Götalandsregionen. Enligt EU:s dataskyddsförordning har du rätt att kostnadsfritt få ta del av de uppgifter om dig som hanteras i projektet, och vid behov få eventuella fel rättade. Du kan också begära att uppgifter om dig raderas samt att behandlingen av dina personuppgifter begränsas. Rätten till radering och till begränsning av behandling av personuppgifter gäller dock inte när uppgifterna är nödvändiga för den aktuella forskningen. Om du vill ta del av uppgifterna ska du kontakta huvudansvarig forskare Emma Hansson, Gröna Stråket 8, 413 45 Göteborg. Tel. 031-3423700. Dataskyddsombud nås på sahlgrenska.universitetssjukhuset.dso@vgregion.se . Om du är missnöjd med hur dina personuppgifter behandlas har du rätt att ge in klagomål till Integritetsskyddsmyndigheten, som är tillsynsmyndighet.

Hur får jag information om resultatet av projektet?

Resultatet kommer att presenteras på gruppnivå i vetenskapliga artiklar. Om du önskar kopia på din utskriva intervju kontaktar du forskningssjuksköterskan (kontaktuppgifter sist i detta dokument).

Försäkring och ersättning

Sedvanlig patientskadeförsäkring gäller. Ingen ersättning utgår för deltagande i projektet.

Deltagandet är frivilligt

Ditt deltagande är frivilligt och du kan när som helst välja att avbryta deltagandet. Om du väljer att inte delta eller vill avbryta ditt deltagande behöver du inte uppge varför, och det kommer inte heller att påverka din framtida vård eller behandling.

Om du vill avbryta ditt deltagande ska du kontakta den ansvariga för projektet (se nedan).

Ansvariga för projektet

Ansvariga för projektet är FOU-sjuksköterska Susanne Meyer, susanne.meyer@vgregion.se Lektor Anna Paganini, specialistsjuksköterska, med dr. anna.paganini@vgregion.se, prof.

Emma Hansson, öl, emma.em.hansson@vgregion.se Alla tre nås på: Verksamhet plastikkirurgi, Sahlgrenska universitetssjukhuset, Gröna Stråket 8, 413 45 Göteborg. Tel. 031-3423700.

Samtycke till att delta i projektet

Jag har fått muntlig och/eller skriftlig information om studien och har haft möjlighet att ställa frågor. Jag får behålla den skriftliga informationen.

☐ Jag samtycker till att delta i projektet *Kroppsegen jämfört med implantatbaserad bröstrekonstruktion - en delvis lottad patientpreferensstudie med 5 års uppföljning (GoBreast II)* Intervju kring nöjdhet/missnöjdhet

Plats och datum	Underskrift
	Namnförtydligande

Information till forskningspersoner

Vi vill fråga dig om du vill delta i ett forskningsprojekt. I det här dokumentet får du information om projektet och om vad det innebär att delta.

Vad är det för ett projekt och varför vill ni att jag ska delta?

I detta forskningsprojekt önskar vi undersöka dina upplevelser och erfarenheter kring val av metod vid bröstrekonstruktion. Syftet är att få mer kunskap kring hur man resonerar när man väljer metod och hur vi skulle kunna förbättra denna process för framtida patienter. Du tillfrågas om deltagande då du deltar i gruppen som själv valt metod i studien GoBreast II (preferensgruppen).

Forskningshuvudman för projektet är Västra Götalandsregionen. Med forskningshuvudman menas den organisation som är ansvarig för projektet. Ansökan är godkänd av Etikprövningsmyndigheten, diarienummer för prövningen hos Etikprövningsmyndigheten är 2023-04754-01.

Hur går projektet till?

Ett deltagande innebär att du kommer att kallas för en intervju med frågor kring ditt val av bröstrekonstruktionsmetod. Intervjun kommer att utföras av en psykolog/psykologstudent eller en sjuksköterska eller en läkare.

Intervjun sker vid två tillfällen (en gång i anslutning till att du gjort valet och en gång cirka ett år senare) och tar cirka 2 timmar per intervju och genomförs där du själv önskar (sjukhuset/hemma hos dig/på din arbetsplats) eller online (Zoom eller Teams).

Studiedeltagande innebär inga andra åtaganden från din sida och kommer inte påverka den vård eller det bemötande du får inom sjukvården

Möjliga följder och risker med att delta i projektet

Deltagande i projektet i studien skulle kunna leda till att gamla känslor kring bröstcancer och dess behandling väcks till liv. I fall det visar sig att du behöver ytterligare hjälp att bearbeta dina upplevelser kommer de forskningsansvariga att ombesörja att du remitteras till rätt vårdinstans för att få sådan hjälp.

Vad händer med mina uppgifter?

Projektet kommer att samla in och registrera information om dig.

Intervjuerna kommer att spelas in och sedan skrivas ut (transkriberas) anonymt. Texterna kommer att förvars kodade och inlåsta. Kodlistan kommer att förvaras inlåst och separerad från utskrift från intervjuer. Texterna och inspelningarna kommer att arkiveras i 25 år.

Informationen kommer bara vara tillgänglig för forskningspersonerna och förvaras i kodad form, så den inte kan härledas till dig. Uppgifterna skyddas som journalhandling. För att kunna tolka svaren kommer vi även att använda sedan data kring din behandling den/de operation/er du genomgått samt om orsakerna till att du opererats, ditt hälsotillstånd (t.ex. tobaksanvändning, vikt och längd), ålder vid operationstillfället och den lista över mål med rekonstruktion du kom fram till innan operationen (PEGASUS), som tidigare samlats in inom ramen för GoBreast II.

Dina svar och dina resultat kommer att behandlas så att inte obehöriga kan ta del av dem.

Ansvarig för dina personuppgifter (personuppgiftsansvarig) är Västra Götalandsregionen. Enligt EU:s dataskyddsförordning har du rätt att kostnadsfritt få ta del av de uppgifter om dig som hanteras i projektet, och vid behov få eventuella fel rättade. Du kan också begära att uppgifter om dig raderas samt att behandlingen av dina personuppgifter begränsas. Rätten till radering och till begränsning av behandling av personuppgifter gäller dock inte när uppgifterna är nödvändiga för den aktuella forskningen. Om du vill ta del av uppgifterna ska du kontakta huvudansvarig forskare Emma Hansson, Gröna Stråket 8, 413 45 Göteborg. Tel. 031-3423700. Dataskyddsombud nås på sahlgrenska.universitetssjukhuset.dso@vgregion.se . Om du är missnöjd med hur dina personuppgifter behandlas har du rätt att ge in klagomål till Integritetsskyddsmyndigheten, som är tillsynsmyndighet.

Hur får jag information om resultatet av projektet?

Resultatet kommer att presenteras på gruppnivå i vetenskapliga artiklar. Om du önskar kopia på din utskriva intervju kontaktar du forskningssjuksköterskan (kontaktuppgifter sist i detta dokument).

Försäkring och ersättning

Sedvanlig patientskadeförsäkring gäller. Ingen ersättning utgår för deltagande i projektet.

Deltagandet är frivilligt

Ditt deltagande är frivilligt och du kan när som helst välja att avbryta deltagandet. Om du väljer att inte delta eller vill avbryta ditt deltagande behöver du inte uppge varför, och det kommer inte heller att påverka din framtida vård eller behandling.

Om du vill avbryta ditt deltagande ska du kontakta den ansvariga för projektet (se nedan).

Ansvariga för projektet

Ansvariga för projektet är FOU-sjuksköterska Susanne Meyer, susanne.meyer@vgregion.se Lektor Anna Paganini, specialistsjuksköterska, med dr. anna.paganini@vgregion.se, prof. Emma Hansson, öl, emma.em.hansson@vgregion.se Alla tre nås på: Verksamhet

plastikkirurgi, Sahlgrenska universitetssjukhuset, Gröna Stråket 8, 413 45 Göteborg. Tel. 031-3423700 .

Samtycke till att delta i projektet

Jag har fått muntlig och/eller skriftlig information om studien och har haft möjlighet att ställa frågor. Jag får behålla den skriftliga informationen.

☐ Jag samtycker till att delta i projektet *Kroppsegen jämfört med implantatbaserad bröstrekonstruktion - en delvis lottad patientpreferensstudie med 5 års uppföljning (GoBreast II)* Intervju kring val av operationsteknik

Plats och datum	Underskrift
	Namnförtydligande
