# Supplementary material for: Gothenburg Breast reconstruction (GoBreast) II protocol: a Swedish partially randomised patient preference, superiority trial comparing autologous and implant-based breast reconstruction
Source: BMJ Open. 2024 Jul 17;14(7):e084025. doi: 10.1136/bmjopen-2024-084025 (PMC11256070; doi:10.1136/bmjopen-2024-084025)
Supplement: Supplementary file 5 [file bmjopen-2024-084025.reviewer_comments.pdf]

PEER REVIEW HISTORY

BMJ Open publishes all reviews undertaken for accepted manuscripts. Reviewers are asked to complete a checklist review form (**http://bmjopen.bmj.com/site/about/resources/checklist.pdf**) and are provided with free text boxes to elaborate on their assessment. These free text comments are reproduced below.

ARTICLE DETAILS

**TITLE (PROVISIONAL)**	Gothenburg Breast reconstruction (GoBreast) II protocol: A Swedish partially randomised patient preference, superiority trial comparing autologous and implant-based breast reconstruction
**AUTHORS**	Hansson, Emma; Löfstrand, Jonas; Larsson, Camilla; Uusimaki, Alexandra; Svensson, Karolina; Ekman, Anna; Svensson, Mikael; Paganini, Anna

VERSION 1 – REVIEW

**REVIEWER**	Dean, Nicola<br>Flinders University, College of Medicine & Public Health
**REVIEW RETURNED**	13-Feb-2024

**GENERAL COMMENTS**	OFFICIAL<br><br>This is an important trial, likely to have global impact.<br><br>1) technical language problem - "non-radiated" and "non-irradiated" and likewise "radiated" and "irradiated" are used in consistently. "irradiated" and "non-irradiated" are the commonly used versions.<br><br>2) Minimal important differences in BREAST Q scores. The authors write that MIDs are not published for the BREAST Q reconstruction module. In fact, MIDs for the reconstruction module are now available (published in 2020) and should be used for interpreting results<br><br>Voineskos, Sophocles H., et al. "Giving meaning to differences in BREAST-Q scores: minimal important difference for breast reconstruction patients." Plastic and reconstructive surgery 145.1 (2020): 11e-20e.<br><br>3) As normative values for the BREAST Q have been found to vary between countries, this excellent trial would be further strengthened by an accompanying population based normative study for Swedish women.<br><br>4) The authors did not outline how they would manage participants who unexpectedly required radiotherapy. For example, a woman undergoind mastectomy for DCIS only may have an unexpected cancer found following histological examination. Explaining how these patients will be analysed will be important.<br><br>5) There is clear evidence in the literature that measured outcomes following delayed reconstruction are often better than those following immediate reconstruction. A plan for analysing this

	as a variable or seeking to have a minimum number of each in each analysis group will be important.<br><br>6) In the section explaining biasing factors for provision of different types of breast reconstruction, commercial factors are not mentioned. Marketing of implants to surgeons and pressure by health insurance companies for patients to undergo "cheaper" surgery (even though it may be less cost-effective in the longterm are the largest distorting factors worldwide. I appreciate this may not be the case in Sweden, but should be mentioned in a global publications.<br><br>Well done for bringing this important trial to fruition<br><br>OFFICIAL

**REVIEWER**	Joshi, Shalaka<br>Tata Memorial Hospital, Surgical Oncology
**REVIEW RETURNED**	11-Mar-2024

**GENERAL COMMENTS**	I congratulate the authors on a well-designed and pragmatic study protocol in this area of reconstruction after mastectomy. It is indeed difficult for patients to make this decision. Following are a few comments regarding the study.<br>1) How do the authors plan to take care of differences in reconstructive outcome with respect to years of experience? For example, the surgeon's experience may have direct correlation with the outcome of reconstructive surgery which may also be then correlated to patient reported outcome measures. Since this is a longitudinal study undergoing currently, one may consider incorporating this point.<br>2) It may be a good idea to use the data from this study to design an audiovisual "decision aid" in this situation.

VERSION 1 – AUTHOR RESPONSE

Reviewer: 1
Dr. Nicola Dean, Flinders University, Flinders Medical Centre
Comments to the Author:
This is an important trial, likely to have global impact.
1) technical language problem - "non-radiated" and "non-irradiated" and likewise "radiated" and "irradiated" are used in consistently. "irradiated" and "non-irradiated" are the commonly used versions.

Reply: Thank you for pointing this out. The usage has been modified throughout the manuscript.

2) Minimal important differences in BREAST Q scores. The authors write that MIDs are not published for the BREAST Q reconstruction module. In fact, MIDs for the reconstruction module are now available (published in 2020) and should be used for interpreting results
Voineskos, Sophocles H., et al. "Giving meaning to differences in BREAST-Q scores: minimal important difference for breast reconstruction patients." Plastic and reconstructive surgery 145.1 (2020): 11e-20e.

Reply: We refer to the above mentioned MIDs and the paper in the manuscript. " There are no anchor-based minimal important differences (MIDs) published for BREAST-Q, but the distribution-based MID is 4 for Satisfaction with the breast/s [27]." However, we still believe that anchor-based MIDs are missing for BREAST-Q. Please indicate if we need to clarify our stance further in the manuscript.

3) As normative values for the BREAST Q have been found to vary between countries, this excellent trial would be further strengthened by an accompanying population based normative study for Swedish women.

Reply: We agree with the referee. We have included the reference for population based norms for Swedish women:

Jepsen C, Paganini A, Hansson E. Normative BREAST-Q reconstruction scores for satisfaction and well-being of the breasts and potential donor sites: what are Swedish women of the general population satisfied/dissatisfied with? *J Plast Surg Hand Surg* 2023;58:124-31. doi: 10.2340/jphs.v58.15301 [published Online First: 20231115]

4) The authors did not outline how they would manage participants who unexpectedly required radiotherapy. For example, a woman undergoind mastectomy for DCIS only may have an unexpected cancer found following histological examination. Explaining how these patients will be analysed will be important.

Reply: This is a very good point. We have added information in the section on analyses (p. 18).

5) There is clear evidence in the literature that measured outcomes following delayed reconstruction are often better than those following immediate reconstruction. A plan for analysing this as a variable or seeking to have a minimum number of each in each analysis group will be important.

Reply: Equally good point. We have added information in the section on analyses (p. 18).

6) In the section explaining biasing factors for provision of different types of breast reconstruction, commercial factors are not mentioned. Marketing of implants to surgeons and pressure by health insurance companies for patients to undergo "cheaper" surgery (even though it may be less costeffective in the longterm are the largest distorting factors worldwide. I appreciate this may not be the case in Sweden, but should be mentioned in a global publications.

Reply: We have added a comment on this in the discussion (p. 26).

Well done for bringing this important trial to fruition

Reviewer: 2
Dr. Shalaka Joshi, Tata Memorial Hospital, Tata Memorial Hospital
Comments to the Author:
I congratulate the authors on a well-designed and pragmatic study protocol in this area of reconstruction after mastectomy. It is indeed difficult for patients to make this decision. Following are a few comments regarding the study.
1) How do the authors plan to take care of differences in reconstructive outcome with respect to years of experience? For example, the surgeon's experience may have direct correlation with the outcome

of reconstructive surgery which may also be then correlated to patient reported outcome measures. Since this is a longitudinal study undergoing currently, one may consider incorporating this point.

Reply: We have added information regarding that all the operations will be performed by consultant plastic surgeons with a subspecialty in breast reconstruction and a minimum of 5 years of independent experience of the used technique (p. 11).

2) It may be a good idea to use the data from this study to design an audiovisual "decision aid" in this situation.

Reply: That is a very good idea which we will keep in mind as a possible ancillary study. However, it will not be incorporated in the protocol at this point.

Yours sincerely,
Emma Hansson, MD, PhD
